# Prospective Open‐Label Safety Study of Edaravone Dexborneol in Filipino Patients With Acute Ischemic Stroke

**DOI:** 10.1002/brb3.71272

**Published:** 2026-03-10

**Authors:** Zenyros Faith Sabellano, Bonifacio Pedregosa, Laurence Kristoffer Batino, Jose Navarro

**Affiliations:** ^1^ Department of Neurology Jose R. Reyes Memorial Medical Center, National Specialty Center for Brain and Spine Care Manila Philippines; ^2^ Department of Neurology Baguio General Hospital and Medical Center Baguio Benguet Philippines

## Abstract

**Background:**

Edaravone dexborneol is a neuroprotective agent combining the free‐radical scavenging properties of edaravone and the anti‐inflammatory, blood–brain barrier–modulating effects of dexborneol. While international trials have demonstrated favorable safety and potential clinical benefit, data in Filipino patients remain lacking. This study evaluated the real‐world safety and tolerability of edaravone dexborneol among Filipino patients with acute ischemic stroke.

**Methods:**

This was a single‐center, open‐label, single‐arm, prospective study conducted under a compassionate‐use program. Patients aged 18–80 years with clinically confirmed acute ischemic stroke within 48 h of onset received edaravone dexborneol (30 mg edaravone + 7.5 mg dexborneol) twice daily for 14 days. Safety monitoring continued until Day 20. Treatment‐emergent adverse events (TEAEs) and serious adverse events (SAEs) were classified using the International Council for Harmonisation (ICH E2A) criteria, and causality was assessed using the World Health Organization–Uppsala Monitoring Centre (WHO–UMC) categories.

**Results:**

Twenty‐seven of 29 enrolled patients completed the 14‐day regimen. Mean age was 55.7 ± 12.8 years; 66.7% were male. Overall, 15 patients (55.6%) experienced at least one TEAE, most commonly headache (29.6%), which occurred on Day 0 and resolved within 0.7 ± 1.2 days. Elevated liver enzymes were observed in 25.9% for aspartate transaminase (AST) as well as for alanine aminotransferase (ALT), generally mild and self‐limited. One patient developed both moderate transaminitis and acute kidney injury, attributed to concurrent antituberculosis therapy rather than the study drug. No treatment discontinuations, life‐threatening reactions, or deaths occurred.

**Conclusion:**

Edaravone dexborneol demonstrated a favorable safety profile in Filipino patients with acute ischemic stroke, with adverse events (AE) predominantly mild, transient, and reversible. These real‐world findings align with international data and support their potential incorporation into acute stroke management pathways in the Philippines.

## Graphical abstract

This prospective, single‐arm study evaluated the real‐world safety of edaravone dexborneol in Filipino patients with acute ischemic stroke treated within 48 h of symptom onset. Adverse events were predominantly mild and transient, with headache and reversible liver enzyme elevations being the most common. No treatment discontinuations, life‐threatening reactions, or deaths occurred, supporting a favorable safety and tolerability profile in this population.

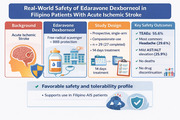

## 1 Introduction

Ischemic stroke, resulting from focal cerebral ischemia and subsequent interruption of cerebral blood flow, remains a leading cause of death and long‐term disability worldwide, accounting for approximately 85% of all stroke cases (Go et al. 2014; World Health Organization 2004). The global burden continues to rise, particularly in low‐ and middle‐income countries such as the Philippines, where younger populations are increasingly affected, reflecting disparities in healthcare access, preventive strategies, and rehabilitation resources (Navarro et al. 2014).

Decades of research have elucidated the complex cellular, molecular, and biochemical cascades that underlie ischemic injury. The ischemic cascade involves oxidative stress, excitotoxicity, inflammation, and apoptosis‐ processes that continue until reperfusion is achieved (Pandian and Sudhan 2013; Minematsu et al. 2012; Yamaguchi et al. 1995; Yamaguchi et al. 2006). While intravenous thrombolysis remains the standard of care for eligible patients (National Institute of Neurological Disorders and Stroke rt‐PA Stroke Study Group 1995; Heiss et al. 1998), its utilization in developing countries remains limited, underscoring the need for adjunctive neuroprotective therapies to reduce secondary neuronal injury and improve outcomes (Heiss et al. 1998; Demchuk et al. 1999; Xu et al. 2013).

Edaravone, a potent free‐radical scavenger, was the first pharmacologic neuroprotectant approved for acute ischemic stroke in Japan. It mitigates oxidative damage by inhibiting lipid peroxidation, preserving neuronal and endothelial integrity, and reducing cerebral edema and delayed neuronal death (Pandian and Sudhan 2013; Minematsu et al. 2012; Yamaguchi et al. 1995; Yamaguchi et al. 2006). Dexborneol, a bicyclic monoterpene with anti‐inflammatory and blood–brain barrier–modulating properties, enhances the penetration and efficacy of coadministered agents (Almeida et al. 2013; Liu et al. 2011).

The combination formulation, edaravone dexborneol, leverages the synergistic antioxidative and anti‐inflammatory mechanisms of both agents. Preclinical studies have demonstrated superior neuroprotection compared with edaravone alone (Wu et al. 2014), while a phase II multicenter randomized trial confirmed favorable safety and efficacy profiles in patients with acute ischemic stroke (Xu et al. 2019). Subsequent studies, including the ECCS‐AIS trial, have shown comparable or improved neurological outcomes with edaravone‐based regimens (Mitta et al. 2012; Feng et al. 2011).

Beyond ischemic stroke, edaravone has shown benefit in various oxidative stress–related conditions such as intracerebral hemorrhage, hypertension, atherosclerosis, diabetes, myocardial infarction, and heart failure (Ramaiah and Yan 2013; Santos 2016; Kongbunkiat et al. 2016; Navarro 2005; Tsujita et al. 2004; Higashi et al. 2006). Combination therapies with thrombolytics and antiplatelet agents have further enhanced recanalization and recovery rates, supporting edaravone's integration into Japanese acute stroke guidelines (Yagi et al. 2009; Kern et al. 2013).

Given the paucity of local data, particularly in the Filipino population, evaluating the safety and tolerability of edaravone dexborneol is essential for guiding clinical adoption. Previous trials have reported a favorable safety profile, with mild adverse events (AEs) such as transient transaminase elevation and hypokalemia, all of which were reversible with continued treatment (Nanjing Simcere Dongyuan Pharmaceutical Co. (China) n.d.; Nanjing Simcere Dongyuan Pharmaceutical Co. (China) n.d.; Otomo 2003; Sharma et al. 2011). This study, therefore, aims to assess the real‐world safety of edaravone dexborneol among Filipino patients with acute ischemic stroke, contributing to regional pharmacovigilance and evidence‐based stroke management.

## 2 Methods

### 2.1 Study Design and Endpoints

#### 2.1.1 Study Design

This was a single‐center, prospective, open‐label, single‐arm study evaluating the safety of edaravone dexborneol in Filipino patients with acute ischemic stroke. The study included a 14‐day treatment period and a follow‐up period extending to Day 20.

#### 2.1.2 Study Endpoints

Adverse events (AEs) were classified according to the International Council for Harmonisation (ICH E2A) definitions. A treatment‐emergent adverse event (TEAE) was defined as any AE that newly appeared or worsened in severity following initiation of edaravone dexborneol therapy.  Serious adverse events (SAEs) were defined as events that resulted in death, were life‐threatening, required or prolonged hospitalization, led to persistent or significant disability/incapacity, or were otherwise considered medically important.

Causality of each AE was assessed using the World Health Organization–Uppsala Monitoring Centre (WHO–UMC) standardized categories. To minimize subjectivity, assessments were performed by the principal investigator and independently reviewed by the study monitor, composed of vascular neurologists, in accordance with WHO–UMC guidance.

#### 2.1.3 Primary Endpoint

The primary endpoint was the incidence, type, and severity of TEAEs associated with edaravone dexborneol administration during the 14‐day treatment period among Filipino patients with acute ischemic stroke.

#### 2.1.4 Secondary Endpoints

The following are the secondary endpoints:

1. Incidence and characterization of SAEs based on ICH E2A criteria.
2. Causality assessment of each AE according to WHO–UMC standardized categories (certain, probable/likely, possible, unlikely, conditional/unclassified, and unassessable).
3. Clinical outcome of each AE/SAE, including recovery, persistence, or death, and the proportion of events requiring treatment discontinuation or dose adjustment, is also based on ICH E2A criteria.

### 2.2 Safety Monitoring

Safety was monitored from the first dose, Day 1 through Day 20. The on‐treatment period comprised Days 1–14, during which participants received edaravone dexborneol twice daily. The post‐treatment follow‐up period (Days 15–20) was used to capture delayed or resolving adverse effects during admission, follow‐up in person, or by telephone. A TEAE was defined as any event that began or worsened in severity between Day 1 and Day 20.

Patients who developed clinically significant laboratory abnormalities, including transaminitis or acute kidney injury (AKI), were promptly referred to the appropriate specialists (gastroenterology or nephrology) for co‐management and supportive care in accordance with institutional protocols.

### 2.3 Study Population

**FIGURE 1:**
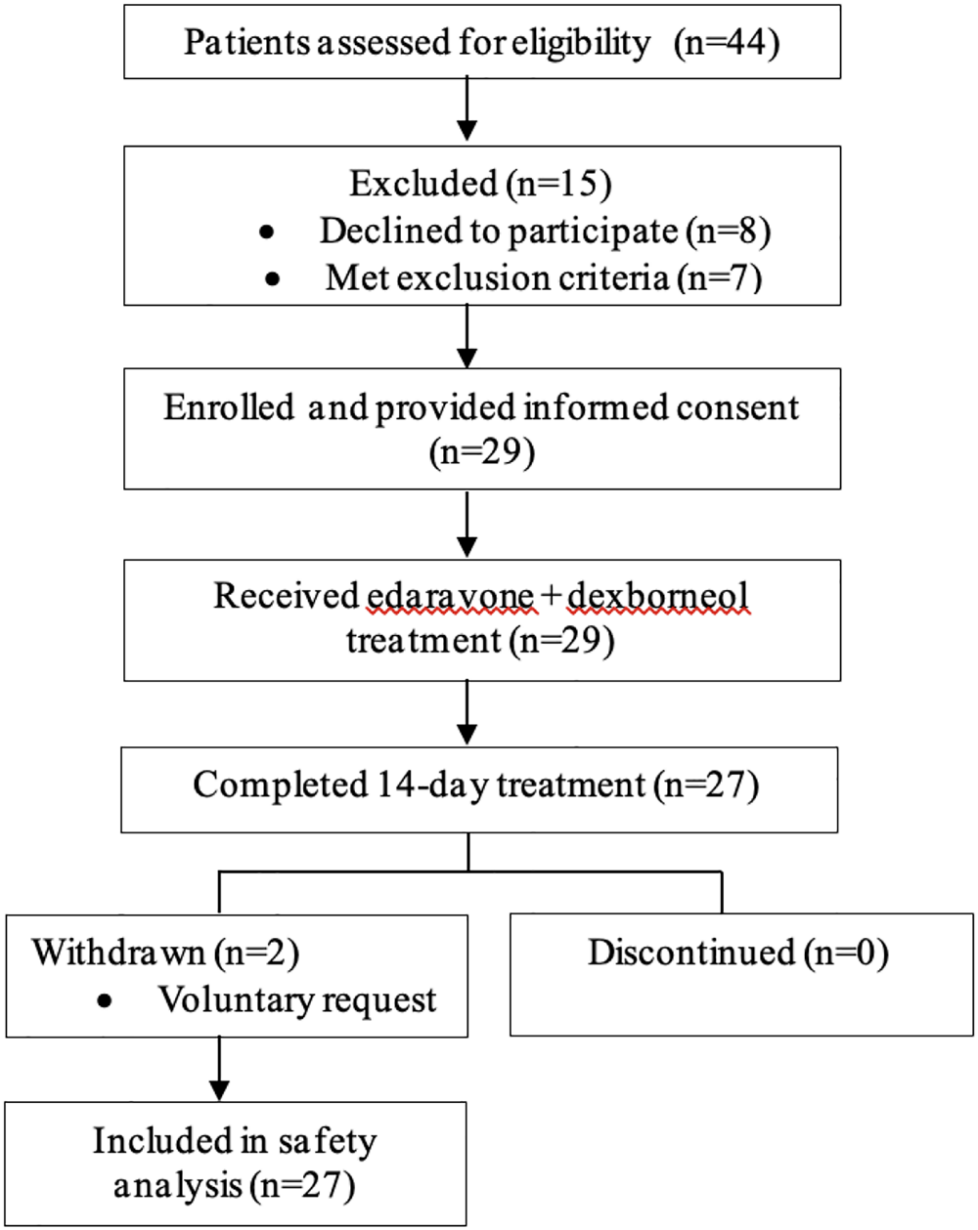
Participant flow diagram.

A total of 29 patients with acute ischemic stroke were enrolled based on the availability of the investigational product provided by the sponsor under a compassionate‐use program. Among these, 27 patients completed the 14‐day treatment course with edaravone dexborneol, while two patients withdrew early due to nondrug‐related reasons (see Figure 1).

#### 2.3.1 Inclusion Criteria

Participants must meet all of the following criteria:

1. patients aged 18–80 years old, male or female;
2. clinically diagnosed as acute ischemic stroke within 48 h stroke onset;
3. premorbid modified Rankin scale ≤ 1;
4. NIH stroke scale of ≤ 25.

#### 2.3.2 Exclusion Criteria

Participants meeting any of the following conditions will be excluded:

1. intracranial hemorrhage or other hemorrhagic disease on CT/MRI (e.g., subdural hematoma, ventricular hemorrhage, or subarachnoid hemorrhage);
2. hypersensitive to edaravone, dexborneol or auxiliary materials;
3. prior receipt of edaravone or dexborneol;
4. systolic blood pressure ≥ 180 mmHg or diastolic blood pressure ≥ 110 mmHg after antihypertensive treatment;
5. serum alanine aminotransferase (ALT) or aspartate transaminase (AST) elevates over three times of upper limit of normal;
6. recent or current serum creatinine is known to exceed 1.5 times the upper limit of normal, or estimated glomerular filtration rate (eGFR) < 60 mL/min;
7. pregnancy, lactation, or planned pregnancy within 90 days;
8. those who cannot complete informed consent or follow‐up treatment due to severe mental disorder or dementia;
9. those with a malignant tumor, severe systemic diseases such as sepsis or unstable vital signs, or predict survival time < 90 days;
10. participate in another interventional clinical study within 30 days before randomization or participate in another interventional clinical study;
11. patients with antibiotics such as cephazolin, piperacillin sodium hydrochloride, and cefuroxime;
12. patients on antiepileptic drugs such as diazepam and phenytoin.

### 2.4 Participant Withdrawal

#### 2.4.1 Reasons for Withdrawal or Termination

Participants were free to withdraw from the study at any time upon request. Investigators could also discontinue a participant's involvement if an SAE, AE, laboratory abnormality, or any medical condition occurred that made continued participation contrary to the patient's best interest. Participation was likewise terminated if a patient was later found to meet, or subsequently developed, an exclusion criterion that precluded further enrollment.

#### 2.4.2 Handling of Withdrawals

Participants who withdrew early were asked to provide the reason for withdrawal voluntarily. They were also requested to grant permission for the use of data collected up to the time of withdrawal for research analysis. If permission was not granted, all data recorded after the date of withdrawal were removed from the study database.

### 2.5 Treatment Protocol

#### 2.5.1 Screening and Informed Consent

All participants were screened for eligibility according to the study's inclusion and exclusion criteria. Informed consent was obtained from each participant or a legally authorized representative before any study procedure. Baseline assessments included demographic data, medical history, physical examination, and vital signs. Laboratory evaluations comprised a complete blood count, serum electrolytes, creatinine, ALT, AST, and a non‐contrast cranial CT scan. Clinical and laboratory data were recorded in standardized observation and case report forms.

#### 2.5.2 Drug Administration

Patients with confirmed ischemic stroke received standard care, including antiplatelet or novel oral anticoagulant therapy based on stroke etiology and high‐dose statin treatment, in accordance with established clinical guidelines. During the treatment phase (Days 1–14), edaravone dexborneol was prescribed by the investigator. It was administered within 48 h of stroke onset. Each dose (30 mg edaravone + 7.5 mg dexborneol; three 5‐mL ampules) was diluted in 100 mL of 0.9% sodium chloride and infused intravenously over approximately 30 min using aseptic technique.

#### 2.5.3 Safety Assessment

AEs and SAEs were monitored throughout the treatment period and recorded in standardized report forms. Demographic data, clinical history, concomitant medications, and treatment details were extracted from the emergency service complex (ESC) physician observation chart (see Appendix A in the Supporting Information) and case report form (see Appendix B in the Supporting Information). Neurological status was assessed using the NIH stroke scale (NIHSS). Causality was evaluated according to the WHO–UMC criteria, classifying events as certain, probable, possible, unlikely, unclassified, or unassessable based on temporal relationship, alternative explanations, and response to drug withdrawal (see Appendix C in the Supporting Information).

### 2.6 Statistical Considerations

#### 2.6.1 Sample Size

A total of 29 patients with acute ischemic stroke were enrolled in the study based on the availability of the investigational product provided by the sponsor under a compassionate‐use program. Among these, 27 patients completed the full 14‐day course of edaravone dexborneol treatment, while two patients withdrew early for nondrug‐related reasons and refused to include their data for analysis.

### 2.7 Statistical Analysis

Descriptive statistics were used for the analysis of AEs. The incidence of each AE or adverse drug reaction was calculated as the proportion of patients who experienced the specific event relative to the total number of participants in the study.

### 2.8 Investigational Product

#### 2.8.1 Formulation, Appearance, Packaging, and Labeling

The investigational product was edaravone dexborneol concentrated solution for injection (10 mg of edaravone and 2.5 mg of dexborneol). The solution was colorless, clear, and contained in borosilicate glass ampules. It is referred to as edaravone dexborneol throughout this paper.

### 2.9 Dosage and Administration

Each dose consisted of 30 mg edaravone + 7.5 mg dexborneol, administered twice daily by intravenous infusion over approximately 30 min.

Before infusion, three 5‐mL ampules were diluted with 100 mL of 0.9% sodium chloride. Treatment was initiated within 48 h of stroke onset and continued for 14 days.

### 2.10 Storage Conditions

Edaravone dexborneol ampules were protected from light and stored at 2°C –8°C in a refrigerator or equivalent controlled environment. The product was received and endorsed to the pharmacy department of the trial site. Cold‐chain integrity during transport was maintained by the designated courier.

### 2.11 Study Drug Accountability

All investigational products were supplied by the sponsor to the study site. The site investigator received the investigational product, verified its quantity and condition, and documented details on the sponsor‐provided Record for Investigational Product form.

This documentation included amounts received, dispensed, and returned, along with dates of each transaction. Dispensing was carried out by the hospital pharmacy. All products were dispensed and stored in accordance with the manufacturer's instructions and within their stated expiry period.

## 3 Results

### 3.1 Baseline Characteristics

Table 1 shows the mean age of participants was 55.7 ± 12.8 years (range, 27–72), and 66.7% were male. Most were married (63.0%), and common vascular risk factors included hypertension (88.9%), smoking history (37.0%), and alcohol use (48.1%). The mean time from stroke onset to admission was 10.5 ± 8.5 h, and the mean baseline NIHSS score was 9.7 ± 5.6, decreasing to 3.8 ± 3.2 at discharge. Good functional outcome (mRS 0–2) was achieved in 24 patients (88.9%), while three (11.1%) had poor outcomes; no deaths occurred during the study monitoring period.

**TABLE 1:** Baseline characteristics of the study population, *N* = 27.

Characteristic	*n* (%) or mean ± SD	Range
Demographics		
Age (years)	55.7 ± 12.8	27–72
Sex		
Male	18 (66.7)	
Female	9 (33.3)	
Civil status		
Single	7 (25.9)	
Married	17 (63.0)	
Widowed	3 (11.1)	
Lifestyle factors		
Smoking history	10 (37.0)	
Alcohol use	13 (48.1)	
Illicit drug use	1 (3.7)	
Comorbidities		
Hypertension	24 (88.9)	
Diabetes mellitus	6 (22.2)	
Dyslipidemia	5 (18.5)	
Coronary artery disease	7 (25.9)	
Atrial fibrillation	6 (22.2)	
History of stroke	1 (3.7)	
Pulmonary tuberculosis	1 (3.7)	
Clinical data		
Time from stroke onset to admission (h)	10.48 ± 8.46	0.83–28.00
Baseline NIHSS^a^	9.67 ± 5.62	2–22
Discharge NIHSS^a^	3.78 ± 3.20	0–12
mRS^b^ on discharge		
Good outcome (0–2)	24 (88.9)	
Poor outcome (3–5)	3 (11.1)	
Death	0	
Systolic blood pressure (mmHg)	144.8 ± 22.1	100–200
Diastolic blood pressure (mmHg)	88.2 ± 12.4	60–120
Laboratory parameters		
Admission		
Serum creatinine (µmol/L)	88.2 ± 35.8	33–196
ALT (U/L)	26.9 ± 12.8	15.6–75.6
AST (U/L)	24.4 ± 14.2	10.6–79.3
Discharge		
Serum creatinine	80.9 ± 37.2	37.5–177
ALT (U/L)	96.0 ± 279.8	15.7–1488
AST (U/L)	67.4 ± 94.3	11.4–460
Given rt‐PA	10 (37.0)	
Stroke subtype		
Large artery atherosclerosis	7 (25.9)	
Cardioembolic	6 (22.2)	
Lacunar	14 (51.9)	
Imaging modality used (admission)		
Cranial CT plain	27 (100)	
Cranial MRI	0	
Location of infarct		
Cortical	13 (48.1)	
Subcortical	13 (48.1)	
Posterior circulation	1 (3.8)	
Concomitant medications		
Antiplatelet agents	21 (77.8)	
Anticoagulants	6 (22.2)	
Statins	27 (100)	
Antihypertensives	27 (100)	
Anti‐Koch's medications	1 (3.7)	

Abbreviations: ALT = alanine aminotransferase, AST = aspartate aminotransferase.

^a^ National institutes of health stroke scale.

^b^ Modified Rankin scale.

At baseline, mean systolic and diastolic blood pressures were 144.8 ± 22.1 mmHg and 88.2 ± 12.4 mmHg, respectively. Laboratory values were within normal limits on admission and showed transient elevations in liver enzymes at discharge (mean ALT = 96.0 ± 279.8 U/L; AST = 67.4 ± 94.3 U/L).

The most common stroke subtype was lacunar infarction (51.9%), followed by large‐artery atherosclerosis (25.9%) and cardioembolic stroke (22.2%). All patients underwent non‐contrast cranial CT on admission. Concomitant medications included antiplatelet agents (77.8%), anticoagulants (22.2%), statins (100%), and antihypertensive medications (100%), with one patient with anti‐Koch's medication during admission.

### 3.2 AEs and Safety Endpoints

Table 2 shows the AEs and safety outcomes of the drug administration. Overall, 15 of 27 patients (55.6%) experienced at least one TEAE. Of these, 14 (93.3%) had a single event and one (6.7%) had multiple events. The most frequently reported TEAE was headache (29.6%), all occurring on Day 0, mostly of moderate intensity and resolving within a mean of 0.7 ± 1.2 days.

**TABLE 2:** Summary of adverse events and safety endpoints, *N* = 27.

Endpoint	Variable/category	*n* (%)	Range
Primary endpoint	**Treatment‐emergent adverse events (TEAEs)** ^a^	**15 (55.6)**	Patients with ≥ 1 TEAE
	Patients with 1 TEAE	14 (93.3)	
	Patients with > 1 TEAE	1 (6.7)	
	Type of TEAE		
	(1) Headache	8 (29.6)	
	• Mild	1 (12.5)	
	• Moderate	7 (87.5)	
	• Severe	0	
	Day started		
	• Day 0	8 (100)	
	Duration	0.7 ± 1.2 days	0–4 days
	(2) Elevated creatinine (Stage 2, moderate)^b^	1 (3.7)	Duration: 0.19 ± 0.96
	(3) Elevated AST^c^	7 (25.9)	Duration: 1.00 ± 2.08
	Grade 1	3 (42.9)	
	Grade 2	3 (42.9)	
	Grade 3	1 (14.2)	
	Grade 4	0	
	(4) Elevated ALT^c^	7 (25.9)	
	Grade 1	4 (57.1)	
	Grade 2	2 (28.6)	
	Grade 3	1 (14.3)	
	Grade 4	0	
Secondary endpoint 1	Serious adverse events (SAEs)^a^	4 (14.8)	
	Transaminitis (≥ grade 2)^c^	4	
	Acute kidney injury	1 (25.0)	Same patient with severe transaminitis
Secondary endpoint 2	Causality^d^		Patients with TEAE (*n* = 15)
	Certain	0	
	Probable/likely	0	
	Possible	14 (93.3)	
	Unlikely	1 (6.7)	
	Conditional/unclassified/unassessable	0	
Secondary endpoint 3	Clinical outcome of AE/SAE^a^		Patients with TEAE (*n* = 15)
	Recovered/resolved	14 (93.3)	
	Persistent	1 (6.7)	
	Required treatment discontinuation	0	

*Note*:One patient experienced multiple SAE‐level laboratory abnormalities (moderate–severe transaminitis and acute kidney injury); SAEs are therefore reported on a patient basis.

Abbreviations: AST = aspartate aminotransferase, ALT = alanine aminotransferase.

^a^ International Council for Harmonisation (ICH E2A).

^b^ Classified according to the kidney disease: improving global outcomes (KDIGO) clinical practice guidelines (2012).

^c^ Severity grading of transaminase elevations was based on the common terminology criteria for adverse events (CTCAE), version 5.0: Grade 1: > 1.5–3.0 x ULN, Grade 2: > 3–5 x ULN, Grade 3 > 5–20 x ULN, Grade 4: > 20 x ULN, Grade 5: death.

^d^ Assessed using the World Health Organization–Uppsala Monitoring Centre (WHO–UMC).

Elevated liver enzymes were observed in seven patients (25.9%) for AST and seven (25.9%) for ALT, typically mild to borderline and self‐limited. One patient developed both moderate transaminitis and AKI, classified as SAEs. This patient has concomitant statin and anti‐Koch's medication. No patient required treatment discontinuation since the SAEs developed after the 14‐day treatment.

In terms of the causality and clinical outcomes, based on WHO–UMC criteria, 14 events (93.3%) were assessed as possibly related to edaravone dexborneol, and one (6.7%) as unlikely related. No events were considered certain, probable, or unassessable. At the end of the observation period, 14 events (93.3%) had fully resolved, and one event (6.7%) persisted without clinical deterioration. There were no deaths or life‐threatening reactions during the study monitoring period.

## 4 Discussion

This prospective, single‐center study represents the first real‐world safety evaluation of edaravone dexborneol among Filipino patients with acute ischemic stroke. The combination was generally well tolerated, with most AEs being mild to moderate, transient, and self‐limited. Importantly, no treatment‐related deaths or treatment discontinuations occurred during the monitoring period, supporting the short‐term safety and tolerability of edaravone dexborneol within a local population treated under compassionate‐use conditions.

The AE profile observed in this cohort, predominantly headache and reversible elevations in liver enzymes, is consistent with findings from large‐scale clinical trials conducted in China, including the TASTE and EDO studies (Xu et al. 2019; Nanjing Simcere Dongyuan Pharmaceutical Co. (China) n.d.; Otomo 2003; Wang et al. 2021). Headache was the most frequently reported TEAE (29.6%), occurring on Day 0 and resolving spontaneously within a short duration, consistent with prior edaravone‐based safety reports (Xu et al. 2019; Nanjing Simcere Dongyuan Pharmaceutical Co. (China) n.d.). This temporal pattern suggests an acute, noncumulative effect and supports the benign nature of this adverse reaction.

Elevations in hepatic transaminases were observed in 25.9% of patients for both AST and ALT and were generally mild, transient, and self‐limited, comparable to the 20%–30% incidence reported in previous studies (Nanjing Simcere Dongyuan Pharmaceutical Co. (China) n.d.; Sharma et al. 2011). Only one patient developed moderate‐to‐severe transaminitis accompanied by AKI, which occurred after completion of the 14‐day edaravone dexborneol treatment course. This patient was receiving concomitant anti‐tuberculosis therapy, including isoniazid and rifampicin, agents well recognized for their hepatotoxic potential and association with renal injury (Saukkonen et al. 2006; Wu et al. 2014). The temporal relationship and known toxicity profiles suggest that these abnormalities were more plausibly attributable to concomitant drug exposure rather than a direct effect of edaravone dexborneol. This finding highlights the importance of careful medication reconciliation and close laboratory monitoring when administering edaravone dexborneol alongside other potentially hepatotoxic or nephrotoxic agents.

It is also noteworthy that all patients in this cohort were receiving statin therapy, which has been independently associated with mild, asymptomatic elevations in liver enzymes in a small proportion of users (Averbukh and Wu 2021). This background exposure may have contributed to hepatic enzyme fluctuations in some patients, underscoring the multifactorial nature of laboratory abnormalities in acute stroke populations receiving multiple medications.

Although randomized, double‐blind, controlled trials remain the gold standard for establishing drug efficacy and safety, real‐world observational studies provide complementary post‐approval data, particularly in populations underrepresented in pivotal trials. The efficacy and safety of edaravone dexborneol in acute ischemic stroke have been demonstrated in phase III randomized intravenous and sublingual studies that formed the basis for regulatory approval (Wu et al. 2014; Xu et al. 2019; Mitta et al. 2012; Wang et al. 2021). Since its introduction into clinical practice, edaravone dexborneol has reportedly been administered to more than 3.0 million patients worldwide, further supporting its post‐marketing safety profile (Nanjing Simcere Dongyuan Pharmaceutical Co. (China) n.d.; Sharma et al. 2011; Wang et al. 2021).

Although efficacy outcomes were not a primary objective of this study, the observed improvement in neurological severity—from a mean baseline NIHSS of 9.7–3.8 at discharge—and the high proportion of patients achieving good functional outcomes suggest favorable short‐term neurological recovery during hospitalization. These findings should be interpreted descriptively but are concordant with prior evidence suggesting potential additive neuroprotective effects of edaravone dexborneol when used alongside standard reperfusion and medical therapies (Wu et al. 2014; Xu et al. 2019; Mitta et al. 2012). The mechanistic synergy between edaravone's antioxidative properties and dexborneol's anti‐inflammatory and blood–brain barrier–modulating effects provides biological plausibility for these observations (Pandian and Sudhan 2013; Minematsu et al. 2012; Yamaguchi et al. 1995; Yamaguchi et al. 2006; Almeida et al. 2013; Liu et al. 2011).

Because intravenous rt‐PA was administered to a subset of patients, a comparative analysis was performed to assess its potential confounding effect on safety and short‐term neurological outcomes. No statistically significant differences were observed between rt‐PA and non‐rt‐PA groups with respect to TEAE, SAE, liver enzyme elevations, AKI, or discharge NIHSS scores (Table S1). These findings suggest that the observed safety profile of edaravone dexborneol was not materially influenced by concomitant thrombolytic therapy, although the small sample size limits definitive conclusions.

The broader clinical development and regulatory trajectory of edaravone dexborneol further contextualize the present findings. The granting of breakthrough therapy designation by the United States Food and Drug Administration reflects regulatory recognition of the drug's potential benefit in a serious condition with unmet therapeutic needs (Nanjing Simcere Dongyuan Pharmaceutical Co. (China) n.d.; Otomo 2003). In this context, the present study contributes incremental real‐world safety data by extending existing trial and post‐marketing evidence to a Filipino population.

Several limitations should be acknowledged. The small cohort size and single‐arm design limit causal inference and the detection of rare or delayed AEs. Laboratory follow‐up was restricted to short‐term observation, and functional outcomes were not powered for efficacy analysis. Nonetheless, the concordance of these findings with prior phase III trials and extensive post‐marketing experience supports the robustness of the observed safety profile and provides a rationale for larger, multicenter post‐marketing studies in Southeast Asian populations (Wang et al. 2021).

## 5 Conclusion

Edaravone dexborneol demonstrated a well‐tolerated safety profile among these Filipino patients with acute ischemic stroke, with AEs predominantly mild, transient, and self‐limited, and could also be explained by disease or other concomitant drugs. These real world findings align with the data from China (Wang et al. 2021; Li et al. 2022) and support its potential incorporation into acute stroke management pathways in the Philippines.

No treatment discontinuations occurred during the observation period. One patient developed moderate transaminitis with concomitant AKI, which was attributed to concurrent antituberculosis therapy rather than the study medication—highlighting the importance of assessing comorbid treatments when determining causality. Overall, the safety profile observed in this cohort closely parallels findings from international trials, supporting the potential integration of edaravone dexborneol into acute stroke management in the Philippines.

## Author Contributions

Zenyros Faith Sabellano conceptualized and designed the study, led data acquisition and analysis, interpreted the results, and drafted the initial manuscript. Bonifacio II C. Pedregosa contributed to study conception and design, provided critical clinical and methodological input, and critically revised the manuscript for important intellectual content. Laurence Kristoffer J. Batino contributed to data collection and interpretation, assisted in manuscript drafting, and participated in critical review of the manuscript. Jose C. Navarro provided overall supervision of the study, contributed to study design and interpretation of findings, and critically revised the manuscript for intellectual content. All authors reviewed and approved the final version of the manuscript and agree to be accountable for all aspects of the work.

## Funding

This study was supported by Conjug8 Corporation, which provided the investigational product (edaravone dexborneol) under a compassionate‐use arrangement. The sponsor had no role in data analysis, interpretation, manuscript writing, or the decision to submit this work for publication.

## Ethics Statement

The study was conducted in accordance with the International Council for Harmonisation–Good Clinical Practice (ICH–GCP) guidelines, the ethical principles of the Declaration of Helsinki (see Supplementary Appendix D), and all applicable local regulations. The Institutional Review Board/Ethics Committee reviewed and approved the protocol and informed consent form before study initiation. Participants were informed of the study objectives, procedures, potential risks and benefits, and their right to withdraw at any time. The sponsor provided the investigational product and covered all study‐related costs, including management of any trial‐related injury.

## Conflicts of Interest

The authors declare no conflicts of interest.

## Supporting information




**Supplementary Material**: brb371272‐sup‐0001‐AppendixA.pdf

**Supplementary Material**: brb371272‐sup‐0002‐AppendixB.pdf

**Supplementary Material**: brb371272‐sup‐0003‐AppendixC.pdf

**Supplementary Material**: brb371272‐sup‐0004‐AppendixD.pdf

**Supplementary Table**: brb371272‐sup‐0005‐TableS1.docx

## Data Availability

The data are not publicly available due to ethical and privacy restrictions but are available from the corresponding author upon reasonable request.
