# Supplementary material for: Prospective Open‐Label Safety Study of Edaravone Dexborneol in Filipino Patients With Acute Ischemic Stroke
Source: Brain Behav. 2026 Mar 10;16(3):e71272. doi: 10.1002/brb3.71272 (PMC12973136; doi:10.1002/brb3.71272)
Supplement: Supplementary file 1 — Supplementary Material: brb371272‐sup‐0001‐AppendixA.pdf [file BRB3-16-e71272-s004.pdf]

Supplementary Appendix A

JOSE R. REYES MEMORIAL MEDICAL CENTER
Rizal Avenue, Sta. Cruz, Manila

ESC BLOTTER SHEET

Triage Category: ( ) Emergent ( ) Urgent ( ) Non-Urgent
Medico-legal: ( ) Yes ( ) No

Department: ______________ ( ) AM
Date/Time of Arrival: __________ ( ) PM

PATIENT INFORMATION

Name (Last Name, Given Name, Middle) | Hospital Number:

Age/Sex/Civil Status | Date of birth | Religion | Nationality/Language

Address: | Brgy No.: | Companion's Name: | Contact Number:

Referred by: ( ) Self ( ) OPD ( ) Private MD ( ) Hospital

Mode of Arrival: ( ) Ambulance ( ) Walk-in ( ) Private Vehicle ( ) Police escort

Historian: ( )Patient ( ) Relative ( ) Friend ( ) Other ( ) No Historian Patient

VITAL SIGNS: BP | HR: | RR: | T: | Oxygen Saturation: | Weight:

CHIEF COMPLAINT

HISTORY OF PRESENT ILLNESS

REVIEW OF SYSTEMS

GENERAL	*EYE*	*ENT*	*CV*	*RESP*
( )Fever<br>( )Chills<br>( )Weakness<br>( ) Nausea	( )Redness ( )Diplopia<br>( )Blurred vision<br>( ) Itching<br>( )Loss of vision	( )Congestion ( )Epistaxis<br>( )Sore throat<br>( ) Hoarseness ( )Ear ache<br>( ) Ear discharge	( )Chest pain<br>( )Palpitations<br>( )Orthopnea<br>( )Pedal edema ( )PND	( )DOB ( ) Cough<br>( )Sputum<br>( )Hemoptysis<br>( )Wheezing
GI<br>( )Abdominal Pain<br>( )Hematochezia<br>( )Hematemesis<br>( ) Vomiting ( ) Melena<br>( ) Constipation	*GU*<br>( )Dysuria ( )Frequency<br>( )Nocturia<br>( )Vaginal bleeding<br>( )Vaginal Discharge	*NEURO*<br>( )Numbness ( )Headache<br>( )Unsteady gait<br>( )Seizure<br>( )loss of consciousness	*MS*<br>( )Neck pain ( )Back pain<br>( )Hip pain ( )Joint pain<br>( )Shoulder pain	*SKIN*<br>( )Rash<br>( )Breast asses<br>( )Brest discharge<br>( )Swelling
PSYCH<br>( )Anxious<br>( )Hallucination<br>( )Stress<br>( )Not sleeping	OTHERS	*ALL SYSTEMS REVIEWED*<br>Negative<br>All others systems negative<br>Incomplete due to:<br>Loss of consciousness/Intubated/Exposure to toxic Chemicals		

MEDICAL HISTORY

PAST MEDICAL/SURGICAL	MEDICATIONS	FAMILY	SOCIAL
( )None ( )PTB ( )Diabetes ( )Asthma<br>( )Cardiac ( )OR ( )Hypertension<br>( )ICU Admission ( )Allergies		( )None ( )PTB<br>( )Diabetes ( )Asthma<br>( )Cardiac ( )Cancer<br>( )Hypertension ( )Allergies	( )Smoker __________<br>( )Alcoholic beverage drinker<br>( )Illicit drug use
Residents on Duty:		Date:	Time:

PHYSICAL EXAMINATION

Initial Assessment:

DIAGNOSTICS

( )CBC ( ) Urinalysis ( ) Fecalysis ( )RBS ( ) Pregnancy Test<br>( ) Na ( ) K ( ) Cl ( )Ca ( ) Creatinine<br>( ) ABG ( ) Protime ( ) PTT ( )Troponin I ( )Troponin T<br>( )CPK MB ( ) CPK MM ( ) CPK Total<br>( )Others ____________	( )12-L-ECG	
		Specify
	( ) X-ray	
	( ) CT scan / MRI	
	( ) UTZ	

ESC BLOTTER SHEET

DISPOSITION			
**Physical Examination**			
**VITAL SIGNS: BP: HR: RR: T: Oxygen Saturation:**			
Date:<br>Time:	( ) Treated and Discharged<br>( ) Admit<br>( ) Absconded<br>( ) Transfer of Service	( ) Home Against Medical Advice	( ) Transfer of Hospital Choice<br>( ) Self conduction ( )Ambulance conduction
**Admitting Diagnosis/Discharge Diagnosis:**<br><br>Number of recuperation days (*if applicable*):			

DISCHARGE PLAN		
a. Medications	b. Special Instructions	c. Follow-up (Date & Time)

Attending Physician:
