# Supplementary material for: Prospective Open‐Label Safety Study of Edaravone Dexborneol in Filipino Patients With Acute Ischemic Stroke
Source: Brain Behav. 2026 Mar 10;16(3):e71272. doi: 10.1002/brb3.71272 (PMC12973136; doi:10.1002/brb3.71272)
Supplement: Supplementary file 2 — Supplementary Material: brb371272‐sup‐0002‐AppendixB.pdf [file BRB3-16-e71272-s005.pdf]

Study Code:	Randomisation no:				Subject initials:			

CASE REPORT FORM

OPEN LABEL SAFETY STUDY OF EDARAVONE DEXBORNEOL FOR THE TREATMENT OF ACUTE ISCHEMIC STROKE

Study reference number

CLINICAL TRIAL SITE/UNIT: JRRMMC

PRINCIPAL INVESTIGATOR: ZF SABELLANO

Subject Initials: | | | |

Subject Randomisation Number: | | | |

I am confident that the information supplied in this case record form is complete and accurate data. I confirm that the study was conducted in accordance with the protocol and any protocol amendments and that written informed consent was obtained prior to the study.

Investigator's Signature: --

Date of signature:

D	d	m	m	m	y	y	y	y

**Study Code:**	**Randomisation no:**				**Subject initials:**			

Inclusion Criteria

		Yes	No*
**1**	Patients ahed 18-80 years old, male or female		
**2**	Clinically diagnosed as acute ischemic stroke within 48 hours of stroke onset		
**3**	Pre-morbid modified Rankin Scale ≤ 1		
**4**	NIH Stroke Scale ≤ 25		
**5**			

*If any inclusion criteria are ticked no then the patient is not eligible for the study.

Exclusion Criteria

		Yes*	No
**1**	CT indicates ICH such as hemorrhagic stroke, subdural hematoma, IVH, SAH		
**2**	Hypersensitive to Edaravone, Dexborneol, or auxiliary materials		
**3**	Prior receipt of Edaravone or Dexborneol		
**4**	Systolic blood pressure ≥ 180 mmHg or diastolic BP ≥ 110 mmHg after anti-HTN treatment		
**5**	Serum AST and ALT elevated over 3 times of upper limit of normal		
**6**	Recent creatinine is known to exceed 1.5 times the upper limit, or eGFR <60 mL/min		
**7**	Pregnancy, lactation, planned pregnancy within 90 days		
**8**	Cannot consent due to severe mental disorder or dementia		
**9**	Malignant tumor, sepsis, unstable vital signs, or predicted survival time <90 days		
**10**	Patients with antibiotics such as cefazolin, piperacillin sodium hydrochloride, cefuroxime		
**11**	Patients on antiepileptic drugs such as diazepam and phenytoin		

* If any exclusion criteria are ticked yes then the patient is not eligible for the study.

Signature: -- Date:

d	d	m	m	m	y	y	y	y

VISIT 1 (SCREENING)

Date: ______________
DD MMM YYYY

INFORMED CONSENT

Please note: written informed consent must be given before any study specific procedures take place or any current therapy is discontinued for the purposes of participation in this study.

Has the subject freely given written informed consent? Yes [ ] No [ ]

DEMOGRAPHIC DATA

Age (yrs): [ ][ ] Sex: Female [ ] Male [ ]

Height (m): [ ] • [ ][ ]

Weight (Kg): [ ][ ] • [ ]

Body Mass Index (BMI = Wt (kg)/H^2 (M): [ ][ ] • [ ]

CONTACT NUMBER [ ]

SMOKING HABITS

Does the subject smoke or use tobacco products? *Yes [ ] No [ ]

* how many cigarettes per day? [ ][ ]

Other, specify --

ALCOHOL CONSUMPTION

Does the subject consume alcohol? Yes [ ] No [ ]

If yes, how many units per week? [ ][ ]

MEDICATIONS TAKEN

Is the subject currently or previously taking any medication including OTC, vitamins and/or

supplements?	Yes		No	
*Record **all** medication on Concomitant Medications page				

VISIT 1 (SCREENING)

PREVIOUS MEDICAL HISTORY

Is there any relevant medical history in the following systems?

Code	System	*Yes	No		Code	System	*Yes	No
1	Cardiovascular				9	Neoplasia		
2	Respiratory				10	Neurological		
3	Hepato-biliary				11	Psychological		
4	Gastro-intestinal				12	Immunological		
5	Genito-urinary				13	Dermatological		
6	Endocrine				14	Allergies		
7	Haematological				15	Eyes, ear, nose, throat		
8	Musculo-skeletal				00	Other		

*If ***YES*** for any of the above, enter the code for each condition in the boxes below, give further details (including dates) and state if the condition is currently or potentially active. If giving details of surgery please specify the underlying cause. Use a separate line for each condition.

		Currently Active?	
**Code**	**Details (including dates)**	**Yes**	**No**

Signature: -- Date:

d	d	m	m	m	y	y	y	y

Study Code:	Randomisation no:				Subject initials:			

VISIT 1 (SCREENING)

PHYSICAL EXAMINATION (to be carried out by medical staff only)

Code	System	*Abnormal	Normal
1	General Appearance		
2	Heart		
3	Lungs		
4	Abdomen		
5	Extremities		

* If ***ABNORMAL*** enter the code for each condition in the boxes below and give brief details. Please use a separate line for each condition.

Code	Details

VITAL SIGNS

Pulse rate [ ][ ][ ] bpm

Blood pressure (seated) [ ][ ][ ] / [ ][ ][ ] mmHg

ECG

Is the ECG: Normal [ ] Abnormal [ ] **

**Description ______________________________

Retain signed and dated trace in the plastic sleeve at back of CRF

Signature: ------------------------------ Date: [ ][ ][ ][ ][ ][ ][ ][ ][ ]

d d m m m y y y y

Study Code:	Randomisation no:				Subject initials:			

VISIT 1 (SCREENING)

LABORATORY ANALYSIS

Initials

Blood for haematology and biochemistry

Taken by

✓	Repeat Sample Required?	Date Taken (dd mmm yyyy)									
	Haematology										
	Clinical Chemistry										

Please insert a copy of all results in the plastic sleeve at the back of the CRF.

Are all final results: Normal ☐ Abnormal NCS ☐ **Abnormal CS ☐

**Description --

Does any result contradict study entry? *Yes ☐ No ☐

Initials:

*If YES, subject must not continue. Please complete off study page.

NIHSS on admission:

____

NIHSS on discharge:

____

Signature: ------------------------------------ Date:

d	d	m	m	m	y	y	y	y

Study Code:	Randomisation no:	☐☐☐	Subject initials:	☐☐☐

VISIT 1 (SCREENING)

End of Visit Checklist: to be completed by Investigator

		Yes	No
1	Does the subject satisfy the inclusion and exclusion criteria to date?	☐	☐
2	Have all screening procedures been completed?	☐	☐
3	Has the concomitant medication page been completed?	☐	☐
4	Is the subject willing to proceed?	☐	☐

Investigator

	Yes	No
Is the subject to continue?	☐	☐
Has medication been collected from Pharmacy?	☐	☐
Have the dosing instructions been explained to the patient?	☐	☐

Signature: -- Date: ☐☐☐☐☐☐☐☐☐
d d m m m y y y y

If '**Yes**' please:

Complete details of next visit and any other needed instructions on the instruction card.

Give the subject the instruction card

Study Code:	Randomisation no:				Subject initials:			

DAY ___ Date:______________
DD MMM YYYY

PHYSICAL EXAMINATION (to be carried out by medical staff only)			
**Code**	**System**	***Abnormal**	**Normal**
1	General Appearance		
2	Heart		
3	Lungs		
4	Abdomen		
5	Extremities		
*** If any changes from baseline, complete adverse event page.**			

VITAL SIGNS

Pulse rate [ ][ ][ ] bpm

Blood pressure (seated) [ ][ ][ ] / [ ][ ][ ] mmHg

LABORATORY ANALYSIS

Initials

Blood for haematology and biochemistry Taken by [ ]

✓	**Repeat Sample Required?**	**Date Taken (dd mmm yyyy)**										
	Haematology											
	Clinical Chemistry											

Please insert a copy of all results in the plastic sleeve at the back of the CRF.

Are all final results: Normal [ ] Abnormal NCS [ ] **Abnormal CS [ ]

**Description ....

Does <u>any</u> result contradict continuation in the study? *Yes [ ] No [ ]

Signature: ____________________ Date: [ ][ ][ ][ ][ ][ ][ ][ ][ ]
d d m m m y y y y

Study Code:	Randomisation no:	☐☐☐	Subject initials:	☐☐☐

CONCOMITANT MEDICATIONS

Medication	Total Daily Dose	Units	Reason	Start Date *(MM/DD/YYYY)*	Stop Date *(MM/DD/YYYY)*	Continuing
				__/__/____	__/__/____	☐
				__/__/____	__/__/____	☐
				__/__/____	__/__/____	☐
				__/__/____	__/__/____	☐
				__/__/____	__/__/____	☐
				__/__/____	__/__/____	☐
				__/__/____	__/__/____	☐
				__/__/____	__/__/____	☐
				__/__/____	__/__/____	☐

Adverse Events

Has the patient experienced any Adverse Events since signing the Informed Consent? ☐ Yes, specify below ☐ No

Study Code:	Randomisation no:				Subject initials:			

AE no.	**Adverse Event** (diagnosis (if known) or signs/symptoms)	**Start Date** dd/mmm/yyyy **and Time** (24 hour clock)	**Stop Date** dd/mmm/yyyy **and Time** (24 hour clock)	**Outcome** 1=Recovered 2=Recovered with sequelae 3=Continuing 4=Patient Died 5=Change in AE 6=unknown	**Severity** 1=Mild 2=Moderate 3=Severe	**Plausible relationship to Study Drug**	**Action taken with Study Drug** 1=None 2=Dose Reduction Temporarily 3=Dose Reduced 4=Discontinued Temporarily 5=Discontinued	**Withdrawn due to AE?**	**Serious AE (SAE)?**	**If SAE does it require immediate reporting? (see Protocol)?**
		/ / :	/ / :			☐ Yes ☐ No		☐ Yes ☐ No	☐ Yes ☐ No	☐ Yes ☐ No
		/ / :	/ / :			☐ Yes ☐ No		☐ Yes ☐ No	☐ Yes ☐ No	☐ Yes ☐ No
		/ / :	/ / :			☐ Yes ☐ No		☐ Yes ☐ No	☐ Yes ☐ No	☐ Yes ☐ No

Study Code:	Randomisation no: ☐☐☐	Subject initials: ☐☐☐

OFF STUDY FORM

Date Off Study: __ __ / __ __ / __ __ __ __
(MM/DD/YYYY)

Date Last Study Medication Taken: __ __ / __ __ / __ __ __ __
(MM/DD/YYYY)

Reason Off Study (Please mark only the primary reason. Reasons **other than Completed Study** require explanation next to the response)

- ☐ Completed study
- ☐ AE/SAE **(complete AE CRF & SAE form, if applicable)** ____________________
- ☐ Lost to follow-up ____________________
- ☐ Non-compliant participant ____________________
- ☐ Concomitant medication ____________________
- ☐ Medical contraindication ____________________
- ☐ Withdraw consent ____________________
- ☐ Death **(complete SAE form)** ____________________
- ☐ Other ____________________
