# Supplementary material for: Prospective Open‐Label Safety Study of Edaravone Dexborneol in Filipino Patients With Acute Ischemic Stroke
Source: Brain Behav. 2026 Mar 10;16(3):e71272. doi: 10.1002/brb3.71272 (PMC12973136; doi:10.1002/brb3.71272)
Supplement: Supplementary file 3 — Supplementary Material: brb371272‐sup‐0003‐AppendixC.pdf [file BRB3-16-e71272-s003.pdf]

Supplementary Appendix C
World Health Organization–Uppsala Monitoring Centre (WHO–UMC) criteria

**Causality term**	**Assessment criteria**<br>**(All points should be reasonably complied with)**
Certain	Event or laboratory test abnormality, with plausible time relationship to drug intake<br>Cannot be explained by disease or other drugs<br>Response to withdrawal plausible (pharmacologically, pathologically)<br>Event definitive pharmacologically phenomenologically (i.e. an objective and specific medical disorder or a recognized pharmacological phenomenon)<br>Rechallenge satisfactory, if necessary
Probable/Likely	Event or laboratory test abnormality, with reasonable time relationship to drug intake<br>Unlikely to be attributed to disease or other drugs<br>Response to withdrawal clinically reasonable<br>Rechallenge not required
Possible	Event or laboratory test abnormality, with reasonable time relationship to drug intake<br>Could also be explained by disease or other drugs<br>Information on drug withdrawal may be lacking or unclear
Unlikely	Event or laboratory test abnormality, with a time to drug intake that makes a relationship improbable (but not impossible)<br>Disease or other drugs provide plausible explanations
Unclassified or Uncertain	Event or laboratory test abnormality<br>More data for proper assessment needed, or<br>Additional data under examination
Unassessable	Report suggesting an adverse reaction<br>Cannot be judged because information is insufficient or contradictory<br>Data cannot be supplemented or verified
