# Supplementary material for: Prospective Open‐Label Safety Study of Edaravone Dexborneol in Filipino Patients With Acute Ischemic Stroke
Source: Brain Behav. 2026 Mar 10;16(3):e71272. doi: 10.1002/brb3.71272 (PMC12973136; doi:10.1002/brb3.71272)
Supplement: Supplementary file 4 — Supplementary Material: brb371272‐sup‐0004‐AppendixD.pdf [file BRB3-16-e71272-s002.pdf]

Supplementary Appendix D

INFORM CONSENT FORM TEMPLATE FOR CLINICAL STUDIES (English version)

Inform Consent Form for patients with acute ischemic stroke.
Zenyros Faith A. Sabellano, MD, MPH, FPNA
Department of Neurology- Section of Stroke and Vascular Neurology
Conjug8 Corporation
Safety profile of Edaravone Dexborneol

PART I: INFORMATION SHEET

PART I: Information Sheet

Introduction:
I am Zenyros Faith A. Sabellano, an adult neurologist from Jose R. Reyes Memorial Medical Center. I am conducting a study to determine the safety profile of a neuroprotective drug, Edaravone Dexborneol, for the treatment of acute ischemic stroke. I am going to give you information and invite you to be part of this study. You do not have to decide today whether or not you will participate in the research. Before you decide, you can talk to anyone you feel comfortable with about this study. There may be some words that you do not understand. Please ask me to stop as we go through the information and I will take time to explain. If you have questions later, you can ask them from me.

Purpose of the research
The purpose of the study is to determine the safety profile of a neuroprotective drug, Edaravone Dexborneol, for the treatment of acute ischemic stroke, by monitoring any side effects or adverse effects within 14 days of treatment, with monitoring up to day 20.

Type of Research Intervention
This is an open label trial wherein you are aware of the medication being given to you.

Participant selection
I will be inviting diagnosed acute ischemic stroke patients admitted within 48 hours from stroke onset, aged 18-80 years old, male or female, with mild to moderate stroke.

Voluntary Participation
Your participation in this study is entirely voluntary. It is your choice whether to
participate or not. If you choose not to participate in this research project, it will not be taken against you. You may change your mind later and stop participating even if you agreed earlier on.

Information on the Trial Drug: Edaravone Dexborneol

1. This is a safety profile study of the drug. A phase III trial studies has been published examining the safety and efficacy of edaravone dexborneol compared to edaravone in patients with acute ischemic stroke has indicated a positive trend in improving functional outcomes 90 days after administration. However, in the Philippines the utilization of this drug has not been explored including its safety.

2. Edaravone Dexborneol is a neuroprotective agent. Pharmacological research indicates that it has better synergistic effect, making it more effective at protecting the brain from ischemic injury leading to improved functional outcomes 90 days after administration.

The common adverse reaction includes a transient increase in transaminases, elevated creatinine, and hypokalemia.

Procedures and Protocol
Once diagnosis is confirmed clinically and through neuroimaging, the patient will be asked to participate if he/she is willing via informed consent. Prior to the drug infusion, screening laboratory and ancillary tests be taken as well as in the end of the treatment for comparison. The administration of the drug will be given within 48 hours: infusion of Edaravone Dexborneol 30mg/7.5ml dissolved in 100 ml normal saline. This will be given for 2 weeks as inpatient. The patient will be monitored for any adverse events during the administration of the drug. If adverse event or serious adverse event will happen, an attending physician will be available for reporting and management. If the adverse event is judged to be related to the study drug, the study sponsor will be responsible for ensuring that the participant will receive appropriate treatment.

Duration of the Study
The study will last for 1 year, from January to December 2025.

Risks
There will be risks accompanied by participation of patients in the research. Adverse events and serious adverse events will be recorded promptly. Edaravone has been proven to be safe in stroke, where only diarrhea and nausea are the most common reported adverse effects. In combination with dexborneol, the common adverse reaction includes a transient increase in transaminases and hypokalemia. Most side effects recovered with treatment.

Benefits
There are pharmacological researches that examine the safety and efficacy of edaravone dexborneol in patients with acute ischemic stroke and has shown a positive trend in improving functional outcomes 90 days after administration. Moreover, participating in this study, you can contribute to the improvement and upgrade of clinical practice guidelines for the management of acute ischemic stroke.

Compensation
No compensation (in the form of finances) will be received by the respondents upon their participation in the study. This will be emphasized prior to consent.

Confidentiality
The information obtained from you which are needed in this study will be kept confidential. I will make sure that the data will be used only to answer specific questions that are needed to meet the objectives of this study. Any information about you will have an assigned number, not your name in which only the principal researcher knows about it.

Sharing the Results
The result of the study will be presented and be submitted officially to the sponsor, section of stroke and vascular neurology, and IRB. If on evaluation, the result is worth sharing to the health professions, it will be made widely available to this group through publication. However, we will maintain our stand to keep all other information that would disclose your identity entirely confidential.

Right to Refuse or Withdraw
You do not have to participate in this research if you feel not to do so. You may stop participating in the research at any time that you wish without losing any of your rights as a patient.

Who to Contact
If you have any questions you may ask them anytime, even after the study has started. If you wish to ask questions later, you may contact me Zenyros Faith Sabellano at 09171258399 or email at zfsabellano@gmail.com, or the Institutional Review Board of JRRMMC with the following contact details: jrrmmc.irb@gmail.com, telephone number (+632) 87119498 local 214.

Part II: Certificate of Consent
I have read the foregoing information, or it has been read to me. I have had the opportunity to ask questions about it and any questions that I have asked have been answered to my satisfaction. I consent voluntarily to participate as a participant in this research.
Print Name of Participant_________________
Signature of Participant __________________
Date_____________________
Day/Month/Year

If illiterate:
I have witnessed the accurate reading of the consent form to the potential participant, and the individual has had the opportunity to ask questions. I confirm that the individual has given consent freely.
Print name of witness____________________ *AND Thumb print of participant*
Signature of witness _____________________
Date ______________________
Day/month/year

Statement by the researcher/person taking consent
I have accurately read out the information sheet to the potential participant, and to the best of my ability made sure that the participant understands that the following will be done:
1. He/ she will be asked to answer personal information baseline demographics and medical history
2. Screening laboratory tests and ancillary tests be done before and after the treatment such as blood testing and brain CT scan
3.He/she will be given edaravone dexborneol for 14 days while admitted via intravenous infusion and adverse effects be monitored and addressed promptly once present

I confirm that the participant was given an opportunity to ask questions about the study, and all the questions asked by the participant have been answered correctly and to the best of my ability. I confirm that the individual has not been coerced into giving consent, and the consent has been given freely and voluntarily.
A copy of this Informed Consent Form has been provided to the participant.
Print Name of Researcher/person taking the consent______________________________
Signature of Researcher /person taking the consent_________________________________
Date _________________________
Day/month/year
