# Supplementary material for: Prospective Open‐Label Safety Study of Edaravone Dexborneol in Filipino Patients With Acute Ischemic Stroke
Source: Brain Behav. 2026 Mar 10;16(3):e71272. doi: 10.1002/brb3.71272 (PMC12973136; doi:10.1002/brb3.71272)
Supplement: Supplementary file 5 — Supplementary Table: brb371272‐sup‐0005‐TableS1.docx [file BRB3-16-e71272-s001.docx]

# Supplementary Table S1. Comparative Analysis According to rt-PA Use

| Variable | rt-PA Yes (n=10) | rt-PA No (n=17) | p-value |
| --- | --- | --- | --- |
| Age, mean (years) | 57.3 | 54.7 | 0.466 |
| Baseline NIHSS, mean | 10.1 | 9.4 | 0.920 |
| Discharge NIHSS, mean | 3.3 | 4.1 | 0.524 |
| ≥1 TEAE, % | 50.0 | 41.2 | 0.706 |
| ≥1 SAE, % | 80.0 | 70.6 | 0.678 |
| Elevated AST/ALT, % | 80.0 | 70.6 | 0.678 |
| Acute kidney injury, % | 100.0 | 94.1 | 1.000 |
